# Recent HIV testing and self-reported HIV prevalence among men who inject drugs in Afghanistan: a nationwide survey in 2019–2020

**DOI:** 10.1186/s12954-025-01183-2

**Published:** 2025-03-14

**Authors:** Ajmal Sabawoon, Sima Naderi, Said Iftekhar Sadaat, Abdul Rasheed, Alim Atarud, Fatemeh Tavakoli, Hamid Sahrifi, Ali Mirzazadeh

**Affiliations:** 1 Kabul Medical University, Kabul, Afghanistan; 2 Mailman School of Public Health, Columbia University, New York City, NY USA; 3 Institute for Global Health Sciences, University of California, San Francisco, CA USA; 4 Health Commons Solutions Lab, Toronto, ON Canada; 5 Youth Health and Development Organization, Kabul, Afghanistan; 6 Independent Global Health Consult, Kabul, Afghanistan; 7 HIV/STI Surveillance Research Center, WHO Collaborating Center for HIV Surveillance, Kerman University of Medical Sciences, Kerman, Iran

**Keywords:** HIV testing, HIV prevalence, People who inject drugs, Afghanistan

## Abstract

**Background:**

People who inject drugs (PWID) remain at high risk for HIV in many countries, including Afghanistan. Previous reports on HIV testing and prevalence in Afghanistan were published in 2012. This study assessed recent HIV testing and self-reported HIV prevalence among male PWID in Afghanistan from 2019 to 2020.

**Method:**

We visited 374 public venues and hotpots where PWID used to gather and meet their peers across 8 cities in Afghanistan to enroll eligible participants in our study. Using interviews and a survey, our trained interviewers collected data on the demographics, types of drugs, HIV testing history, and self-reported HIV status of the participants. We analyzed the data using the venues and hotpots as clusters to report the percentages of recent HIV tests and self-reported HIV prevalence overall and in subgroups defined by demographic characteristics and locations.

**Results:**

Among the 1385 participants, most were from Kabul city (28.9%), spoke Dari (67.4%), were aged 25–34 years (42.1%), and were married (52.4%). Overall, 70.7% (95% CI 67.6–73.6) (ranging from 20.0% in Kandahar to 99.3% in Mazar-i-Sharif) were tested for HIV within the past 12 months. Among those who had ever been tested for HIV, 20.7% (95% CI 17.8–24.0) (ranging from 0% in Zarang to 63.2% in Kabul) reported being positive for HIV.

**Conclusion:**

Compared with the results of a similar study in 2012, we found a significant improvement in HIV testing coverage among PWID in Afghanistan. The high self-reported HIV prevalence among this group also highlights the need for targeted screening and treatment programs for PWID in Afghanistan, particularly in the cities of Kabul and Jalalabad.

## Introduction

The updated data for HIV and AIDS are limited in Afghanistan. As of 2023, UNAIDS estimates 13,000 (4200–54000) adults and children living with HIV in Afghanistan, with an estimated 1700 (< 500–9200) new infections per year. It is estimated that only 3300 people living with HIV know their HIV status [1].

People who inject drugs (PWID) continue to be at high risk for HIV in many countries, including Afghanistan. Using multiple methods of population size estimation, we estimated 11,506 PWID in 8 study cities and projected approximately 57,000 PWID in Afghanistan [2]. The latest reported HIV prevalence among PWID was 4.4% among PWID in 2012. The prevalence of HIV among PWID varies significantly across cities: 1.0% in Mazar-i-Sharif, 3.1% in Kabul, 18.4% in Herat [3] 0.3% in Mazar, 0.9% in Charikar, 1.0% in Jalalabad, 2.4% in Kabul and 13.3% in Herat [4].

HIV testing plays a crucial role in preventing HIV transmission. Among PWID, preventing HIV is one of the most cost-effective strategies [5]. HIV testing and counseling allow people at risk to make informed decisions to start HIV treatment and to engage more in prevention measures [6, 7]. To break the chain of transmission, early HIV diagnosis is also critical [8]. A previous study among PWID in Kabul was conducted in 2012, when only 2.7% of PWID had ever been tested for HIV [4].

Voluntary Counselling and Testing Centers, HIV care and ART are offered in five provinces (Kabul, Herat, Mazar, Nangarhar and Khost) in Afghanistan [9]. HIV diagnosis in Afghanistan follows the national guideline using three consecutive rapid tests. Those testing positive on all three are referred to antiretroviral therapy (ART) centers, where a confirmatory rapid test is conducted before starting treatment. Only confirmed positive cases receive ART. Since 2016, Afghanistan has implemented a “test and treat” strategy, initiating (ART for all confirmed HIV-positive patients regardless of CD4 count or disease stage.

The most recent data on HIV testing and prevalence among people who inject drugs (PWID) in Afghanistan came from a 2012 survey, more than a decade old. In our study, we examined recent HIV testing and the prevalence of self-reported HIV among male PWID in Afghanistan during 2019–2020. Additionally, we analyzed the associations between various covariates and both recent HIV testing and self-reported HIV prevalence among this population.

## Methods

We analyzed the data that were collected in a cross-sectional survey from 2019 to 2020. The study locations included eight cities (Kabul, Herat, Mazar-I-Sharif, Jalalabad, Kunduz, Faizabad, Kandahar, and Zaranj) to provide relatively representative cross-sections of the regions of Afghanistan. Eligible PWID were anyone aged 18 years and older who had injected any type of drug at least once for nonmedical purposes in the past 12 months. We found only 9 female PWIDs, so the current analysis focused only on male PWIDs (excluding women because of the very small sample size). All participants provided verbal informed consent via procedures approved by the Institutional Review Boards (IRBs) of the Afghanistan National Public Health Institute, Ministry of Public Health (#444899, 12/29/2018), and the University of California San Francisco (#234207, 03/08/2019).

We applied venue-based purposive sampling to enroll PWID. We used key informant mapping to identify locations (venues) where PWID congregate, the days and times they frequent these spots. A total of 217 individuals, including representatives from NGOs, government officials, health authorities, DIC staff, and current or former PWID, were interviewed individually or in focus group discussions. Key informants from across the city identified hotspots, and peak activity hours. Then study staff were trained in a 3-day workshop to conduct interviews and collect data on demographics, drug use behaviors, HIV testing, and HIV self-reported test results. Field supervisors monitored data collection daily, and team leads provided weekly oversight. The details of the study design were presented previously [2].

The data were collected through a structured data collection form. The first part of the form included screening questions assessing the participants’ eligibility, followed by demographic and drug-related questions. Furthermore, the form contained questions about risk behaviors, HIV testing history, and self-reported HIV infection status.

We analyzed the data for two primary outcomes. The first outcome, HIV testing, was categorized as an ordinal variable with three levels: tested within the last 12 months, tested more than 12 months ago, and never tested. For further analysis, we created a binary outcome for recent HIV testing, classifying participants as either having been tested in the last 12 months (yes) or not (no). The second outcome focused on HIV self-reported status among individuals who had ever been tested for HIV, creating a binary variable to distinguish between those who self-reported as HIV-positive and those who reported being HIV-negative (yes/no). Of the participants who had ever been tested for HIV, 172 (15.1%) did not disclose their HIV status; these individuals were excluded from the analysis of self-reported HIV status.

We analyzed the associations between various covariates and both HIV testing and self-reported HIV prevalence using separate bivariable and multivariable logistic regression models to identify significant predictors while adjusting for potential confounders. In the bivariable analyses, each covariate was individually tested for its association with HIV testing and self-reported HIV prevalence, by chi-saure test and by simple logistic regression providing unadjusted odds ratios (ORs). In the multivariable logistic regression models, we included all covariates to estimate adjusted odds ratios (AORs), controlling for the effects of other variables and ensuring robust results. The covariates included in the analyses were the city of study, language spoken (as a proxy for regional and cultural background), age, and marital status. Substance use variables included heroin use, cocaine use, opium use, amphetamine use, and prescribed drug use. Additional covariates were the time of injection (e.g., daytime vs. nighttime) and sexual behaviors, specifically ever having oral or anal sex with other men. These factors were chosen based on their relevance to HIV transmission and testing behaviors, as well as prior evidence in the literature.

## Results

We analyzed the data of 1385 male PWID (Table 1). Most of the participants were from Kabul city (28.9%), spoke Dari (67.4%), were aged 25–34 years (42.1%) and were married (52.4%). Almost all (99.4%) reported using heroin in the previous month. Most participants (72.9%) reported their last injection to be in the previous month, whereas 3.8% reported engaging in oral or anal sex with other men in their lifetime.

**Table 1:** Demographics and risk behaviors of men who inject drugs in urban cities in Afghanistan from 2019–2020, *n* = 1385

Variable	Number	Percent
**Demographic variables**		
**City**		
Kabul	400	28.9
Herat	200	14.4
Mazar-i-sharif	149	10.8
Jalalabad	150	10.8
Kunduz	151	10.9
Faizabad	35	2.5
Kandahar	150	10.8
Zarang	150	10.8
**Language**		
Pashto	435	31.4
Dari	934	67.4
Uzbek	16	1.2
**Age**		
18–24	198	14.3
25–34	583	42.1
35–44	411	29.7
45+	193	13.9
**Marital Status**		
Single	572	41.3
Married and living with his partner	425	30.7
Married but not living with his partner	301	21.7
Separated/divorced/Widowed	87	6.3
**Drug and sexual behaviors**		
Frequent drug use in the last month*		
Heroin	1,376	99.4
Cocaine	22	1.6
Opium	53	3.8
Amphetamines or Methamphetamines	413	29.8
Prescribed drugs	49	3.5
**Time of last injection**		
Within the last 1 month	1,010	72.9
Within the last 3 months	134	9.7
Within the last 12 months	241	17.4
**Ever had oral or anal sex with other men**		
Yes	53	3.8
No	1,332	96.2

* Some participants used more than one drug, and so the total percent exceeded 100%

Among the 1385 male PWID, 70.7% had been tested within the last 12 months, 11.3% had been tested more than 12 months prior, and 18% had never been tested for HIV. The proportion of HIV testing within the last 12 months ranged from 20.0% in Kandahar to 99.3% in Mazar-i-Sharif. The proportion of recent HIV tests within the last 12 months was significantly lower in those who spoke Pashto (65.3%) and those who used opium (37.7%) than in their counterparts (Table 2).

**Table 2:** HIV testing history by demographics and risk behaviors of men who inject drugs in Afghanistan, *n* = 1385

Characteristics	*n*	Tested within the last 12 months	Tested more than a year ago	Never tested	*P* value
		% (95% CI*)	% (95% CI)	% (95% CI)	
Overall	1385	70.7 (67.6, 73.6)	11.3 (9.3, 13.6)	18.0 (15.8, 20.5)	
**City**					
Kabul	400	66.8 (60.9, 72.1)	8.2 (5.8, 11.6)	25.0 (20.0, 30.8)	
Herat	200	73.0 (64.1, 72.1)	14.0 (9.2,20.7)	13.0 (9.1, 18.3)	< 0.001
Mazar-i-sharif	149	99.3 (95.4, 99.9)	0.7 (0.1, 4.6)	0	
Jalalabad	150	98.7 (90.6, 99.8)	0.7 (0.1, 4.8)	0.7 (0.1, 4.8)	
Kunduz	151	68.9 (54.9, 80.1)	20.5 (10.5, 36.1)	10.6 (6.3, 17.2)	
Faizabad	35	94.3 (79.7, 98.6)	2.9 (0.4, 17.1)	2.9 (0.3, 19.8)	
Kandahar	150	20.0 (13.0, 29.5)	36.0 (28.9, 43.7)	44.0 (35.1, 53.3)	
Zarang	150	68.7 (13.0, 29.5)	4.7 (28.9, 43.7)	26.7 (35.1, 53.3)	
**Language**					
Pashto	435	65.3 (60.3, 70.0)	14.3 (11.0, 18.3)	20.5 (16.5, 25.1)	
Dari	934	72.8 (69.2, 76.1)	10.0 (8.0, 12.4)	17.2 (14.7, 20.1)	0.018
Uzbek	16	93.8 (65.8, 99.2)	6.2 (0.8, 34.2)	0	
**Age**					
18–24	198	68.2 (61.0, 74.6)	11.6 (7.7, 17.1)	20.2 (15.2, 26.3)	
25–34	583	71.9 (66.9, 76.4)	10.6 (7.3, 15.2)	17.5 (14.3, 21.2)	
35–44	411	70.3 (65.3, 74.9)	10.9 (8.3, 14.4)	18.7 (15.1, 23.0)	
45+	193	70.5 (63.1, 76.9)	13.5 (9.3, 19.2)	16.1 (11.5, 22.0)	0.883
**Marital Status**					
Single	572	69.2 (64.4, 73.7)	11.4 (8.4, 15.1)	19.4 (16.0, 23.3)	
Married and living with partner	425	68.2 (63.0, 73.0)	12.7 (9.7, 16.5)	19.1 (15.2, 23.6)	
Married not living with partner	301	77.1 (71.8, 81.6)	9.6 (6.7, 13.6)	13.3 (9.8, 17.8)	
Separated/divorced/Widowed	87	70.1 (60.1, 78.5)	9.2 (4.4, 18.1)	20.7 (13.3, 30.8)	0.225
**Heroin use in the last month**					
No	9	not reliable	not reliable	not reliable	
Yes	1376	70.7 (67.7, 73.6)	11.3 (9.4, 13.7)	18.0 (15.7, 20.4)	0.329
**Cocaine use in the last month**					
No	1363	70.8 (67.7, 73.7)	11.2 (9.2, 13.5)	18.0 (15.8, 20.5)	
Yes	22	63.6 (40.1, 82.1)	18.2 (6.2, 42.7)]	18.2 (8.6, 34.5)	0.550
**Opium use in the last month**					
No	1332	72.0 (68.9,74.9)	10.0 (8.1, 12.2)	18.0 (15.7, 20.6)	
Yes	53	37.7 (24.4, 53.3)	43.4 (31.0, 56.7)	18.9 (11.5, 29.4)	< 0.001
**Amphetamines and methamphetamine use in the last month**					
No	972	70.3 (67.0, 73.4)	11.7 (9.8, 14.0)	18.0 (15.7, 20.6)	
Yes	413	71.7 (65.5, 77.1)	10.2 (6.6, 15.3)	18.2 (13.7, 23.6)	0.794
**Prescribed drug use in the last month**					
No	1336	71.0 (67.9, 74.0)	11.3 (9.3, 13.7)	17.7 (15.4, 20.2)	
Yes	49	61.2 (44.8, 75.5)	10.2 (4.5, 21.4)	28.8 (16.8, 44.3)	0.184
**Ever oral or anal sex with other men**					
Yes	53	75.5 (62.3, 85.1)	7.5 (2.9, 18.4)	17.0 (9.1, 29.5)	
No	1332	70.5 (67.4, 73.4)	11.4 (9.4, 13.8)	18.1 (15.9, 20.5)	0.634

*95% Confidence Intervals

Among those who had ever been tested for HIV (1136), 964 reported their HIV status (172 or 15.1% had missing data on self-reported HIV status). Among those 964 individuals who reported their HIV status (Table 3), 20.7% reported having HIV-positive results in the past. The self-reported HIV prevalence ranged from 0% in Zarang to 27.1% in Jalalabad and 63.2% in Kabul. The prevalence of self-reported HIV was significantly greater among male PWID who spoke Pashto (26.5%), were aged 35–44 years (27.1%), were married and lived with their partner (29.6%), did not use opium in the previous month (21.4%), used amphetamine or methamphetamine (37.3%), and reported injection in the previous month (25.6%) than among their counterparts.

**Table 3:** The prevalence of self-reported HIV status by demographics and risk behaviors of men who inject drugs, Afghanistan, *n* = 964

Characteristics	*n*	% (95% CI*)	*P* value
**Overall**	**964**	**20.7 (17.8**,** 24.0)**	
**City**			
Kabul	228	63.2 (50.8, 74.0)	< 0.001
Herat	155	3.2 (1.3, 7.8)	
Mazar-i-sharif	147	1.4 (0.3, 5.3)	
Jalalabad	144	27.1 (20.4, 34.9)	
Kunduz	132	2.3 (0.7, 6.8)	
Faizabad	3	not reliable	
Kandahar	72	8.3 (4.2, 15.9)	
Zarang	83	0	
**Language**			
Pashto	324	26.5 (20.8, 33.2)	0.016
Dari	624	18.3 (14.7, 22.4)	
Uzbek	16	0	
**Age**			
18–24	133	14.3 (14.6, 21.6)	0.009
25–34	413	18.2 (14.6, 22.3)	
35–44	280	27.1 (21.8, 33.2)	
45+	138	21.7 (14.9, 30.6)	
**Marital Status**			
Single	389	16.5 (12.8, 20.8)	< 0.001
Married and living with his partner	301	29.6 (24.5, 35.2)	
Married but not living with his partner	224	19.6 (14.4, 26.2)	
Separated/divorced/Widowed	50	6.0 (1.9, 17.2)	
**Heroin use in the last month**			
No	5	not reliable	0.281
Yes	959	20.6 (17.7, 23.9)	
**Cocaine use in the last month**			
No	948	20.9 (17.9, 24.2)	0.437
Yes	16	12.5 (2.9, 40.6)	
**Opium use in the last month**			
No	927	21.4 (18.3, 24.8)	0.018
Yes	37	5.4 (1.4, 18.5)	
**Amphetamines and methamphetamine use in the last month**			
No	669	13.5 (10.9, 16.6)	< 0.001
Yes	295	37.3 (29.0, 46.4)	
**Prescribed drug use in the last month**			
No	933	21.1 (18.1, 24.5)	0.1094
Yes	31	9.7 (3.3, 25.2)	
**Time of last injection**			
Within the last 1 month	742	25.6 (21.8, 29.8)	< 0.001
Within the last 3 months	71	8.5 (3.9, 17.2)	
Within the last 12 months	151	2.6 (1.0, 6.5)	
**Ever oral and anal sex with other men**			
Yes	38	10.5 (3.2, 29.5)	0.1853
No	926	21.2 (18.2, 24.5)	

* 95% Confidence Intervals

In the multivariable logistic regression analysis for recent HIV tests (Table 4), those PWID who lived at Herat ([Adjusted Odds Ratio] AOR = 2.35; 95% CI: 1.22–4.54), Mazar-i-sharif (AOR = 71.13; 95% CI: 9.35–541.10), Jalalabad (AOR = 42.19; 95% CI: 5.20–342.61), Faizabad (AOR = 12.74; 95% CI: 2.84–57.18) has a higher odds for being tested for HIV recently. While those who lived in Kandahar (AOR = 0.17; 95% CI: 0.08–0.38) [vs. those in Kabul], those injected with opium last month (AOR = 0.38; 95% CI: 0.17–0.88) [vs. not injecting opium during the last month], those injected within the last 12 months (AOR = 0.46; 95% CI: 0.27–0.77) [vs. those injected within the last month], and those who had ever had oral or anal sex with another man (AOR = 0.40; 95% CI 0.20–0.78) has a lower odds for being tested for HIV recently.

**Table 4:** Bivariate and multivariable logistic regression analysis of factors associated with recent HIV tests of men who inject drugs in Afghanistan

Characteristics	Crude				Adjusted			
	OR	95%CI LL	95%CI UL	*P* value	OR	95%CI LL	95%CI UL	*P* value
City**								
Kabul	Ref.				Ref.			
Herat	1.35	0.83	2.19	0.230	2.35	1.22	4.54	0.011
Mazar-i-sharif	73.72	10.15	535.31	< 0.001	71.13	9.35	541.1	< 0.001
Jalalabad	36.86	4.74	286.78	0.001	42.19	5.2	342.61	0.001
Kunduz	1.1	0.58	2.11	0.768	1.39	0.65	2.96	0.397
Faizabad	8.22	1.91	35.42	0.005	12.74	2.84	57.18	0.001
Kandahar	0.12	0.07	0.22	< 0.001	0.17	0.08	0.38	< 0.001
Zarang	1.09	0.65	1.82	0.736	1.29	0.72	2.3	0.389
**Language**								
Pashto	Ref.				Ref.			
Dari	1.42	1.1	1.85	0.008	0.89	0.60	1.34	0.579
Uzbek	7.98	1.06	59.82	0.043	0.99	0.15	6.62	0.994
Age								
18–24	Ref.				Ref.			
25–34	1.19	0.82	1.72	0.349	1.10	0.70	1.74	0.673
35–44	1.11	0.75	1.63	0.612	1.17	0.73	1.88	0.506
45+	1.11	0.70	1.76	0.644	1.41	0.83	2.40	0.204
**Marital Status**								
Single	Ref.				Ref.			
Married and living with partner	0.95	0.7	1.3	0.769	0.91	0.64	1.29	0.587
Married not living with partner	1.49	1.09	2.06	0.014	1.20	0.81	1.79	0.370
Separated/divorced/Widowed	1.04	0.63	1.72	0.869	1.20	0.61	2.36	0.588
**Frequent drug use in the last month**								
**Heroin use in the last month**								
No	Ref.				Ref.			
Yes	1.21	0.3	4.78	0.788	0.72	0.15	3.34	0.670
**Cocaine use in the last month**								
No	Ref.				Ref.			
Yes	0.72	0.27	1.91	0.511	0.75	0.24	2.34	0.620
**Opium use in the last month**								
No	Ref.				Ref.			
Yes	0.24	0.12	0.45	< 0.001	0.38	0.17	0.88	0.023
**Amphetamines and methamphetamine use in the last month**								
No	Ref.				Ref.			
Yes	1.07	0.78	1.46	0.668	1.07	0.73	1.58	0.717
**Prescribed drug use in the last month**								
No	Ref.				Ref.			
Yes	0.64	0.32	1.29	0.216	2.16	0.78	5.97	0.136
**Time of last injection**								
Within the last 1 month	Ref.				Ref.			
Within the last 3 months	0.53	0.35	0.79	0.002	0.68	0.39	1.18	0.167
Within the last 12 months	0.30	0.20	0.43	< 0.001	0.46	0.27	0.77	0.003
**Ever oral or anal sex with other men**								
Yes	Ref.				Ref.			
No	0.78	0.41	1.47	0.435	0.4	0.2	0.78	0.008

* Recent HIV testing is defined as self-report of an HIV test in the last 12 months before the survey

In the multivariable logistic regression analysis for HIV self-report status (Table 5), PWID who lived in Herat (AOR = 0.03; 95% CI: 0.01–0.13) Mazar-i-sharif (AOR = 0.01; 95% CI: 0.00-0.03), Jalalabad (AOR = 0.05; 95% CI: 0.01–0.19), Kunduz (AOR = 0.01; 95% CI: 0.00-0.04) and Kandahar (AOR = 0.03; 95% CI: 0.01–0.05) [vs. those in Kabul]; spoke Dari (AOR = 0.36; 95% CI: 0.15–0.88) [vs. Pashto]; and were injected with opium last month (AOR = 0.13; 95% CI: 0.04–0.51) [vs. not injecting opium during the last month]; and were injected within the last 12 months (AOR = 0.28; 95% CI: 0.09–0.90) [vs. those injected within the last month] has a lower odds for being self-reported HIV positive.

**Table 5:** Bivariable and multivariable logistic regression analysis of factors associated with self-reported HIV-positive status of men who inject drugs, Afghanistan

Characteristics	Crude				Adjusted			
	OR	95%CI		*P* value	OR	95%CI		*P* value
City*								
Kabul	Ref.							
Herat	0.02	0.01	0.06	< 0.001	0.03	0.01	0.13	< 0.001
Mazar-i-sharif	0.01	0.00	0.04	< 0.001	0.01	0	0.03	< 0.001
Jalalabad	0.22	0.12	0.41	< 0.001	0.05	0.01	0.19	< 0.001
Kunduz	0.01	0.00	0.05	< 0.001	0.01	0	0.04	< 0.001
Faizabad	0.29	0.01	5.85	0.419	0.88	0.02	36.16	0.946
Kandahar	0.05	0.02	0.13	< 0.001	0.03	0.01	0.14	< 0.001
Zarang	Not Reliable*							
Language**								
Pashtoo	Ref.							
Dari	0.62	0.4	0.95	0.029	0.36	0.15	0.88	0.025
Uzbek	Not Reliable							
**Age**								
18–24	Ref.				Ref.			
25–34	1.33	0.78	2.27	0.291	1.19	0.57	2.45	0.643
35–44	2.24	1.24	4.03	0.008	1.55	0.67	3.59	0.302
45+	1.67	0.88	3.17	0.120	0.84	0.33	2.14	0.715
**Marital Status**								
Single	Ref.				Ref.			
Married and living with his partner	2.13	1.47	3.1	< 0.001	1.46	0.84	2.54	0.174
Married but not living with partner	1.24	0.82	1.88	0.307	1.2	0.61	2.33	0.596
Separated/divorced/Widowed	0.32	0.1	1.09	0.069	0.75	0.2	2.87	0.672
**Heroin use in the last month**								
No	Ref.				Ref.			
Yes	0.39	0.07	2.31	0.298	0.75	0.05	10.75	0.828
**Cocaine use in the last month**								
No	Ref.			Ref.	Ref.			
Yes	0.54	0.11	2.62	0.444	1.66	0.29	9.36	0.565
**Opium use in the last month**								
No	Ref.				Ref.			
Yes	0.21	0.05	0.87	0.031	0.13	0.04	0.51	0.003
**Amphetamines and methamphetamine use in the last month**								
No	Ref.				Ref.			
Yes	3.83	2.40	6.09	< 0.001	0.56	0.23	1.36	0.202
**Prescribed drug use in the last month**								
No	Ref.				Ref.			
Yes	0.4	0.1300	1.28	0.122	1.82	0.32	10.32	0.496
**Time of last injection**								
Within the last 1 month	Ref.			Ref.	Ref.			
Within the last 3 months	0.27	0.12	0.62	0.002	0.61	0.26	1.44	0.258
Within the last 12 months	0.08	0.03	0.21	< 0.001	0.28	0.09	0.9	0.032
**Ever oral and anal sex with other men**								
Yes	Ref.			Ref.	Ref.			
No	2.28	0.65	8.02	0.197	2.55	0.79	8.22	0.118

* Zarang city was excluded due to a small number of respondents with HIV positive status ** Uzbek ethnic group was excluded

## Discussion

Our results revealed that approximately 70% of male PWID had been tested for HIV in the last 12 months before the survey. Compared with previous studies (2012), HIV testing coverage has increased significantly across several cities in Afghanistan. However, HIV testing was significantly lower among male PWID living in Kabul and Kandahar and among those who injected opium and those who reported having sex with other men. These areas and subpopulations should be prioritized for more targeted HIV testing campaigns or programs.

Another important finding of our study was that among those male PWID who had ever been tested for HIV, one in five reported a positive HIV test result. While a direct comparison of our finding on self-reported HIV prevalence to those results from the previous study (2012) is not possible, our findings suggested an increase in the HIV prevalence (from 4.4% in 2012 to 20.7% in 2019-20) among male PWID. The HIV prevalence estimates in our study (2019-20) were based on self-reported data, which may partly reflect improvements in HIV coverage and case-finding efforts. Additionally, those who lived in Kabul, spoke Pashto, were married/lived with a partner, aged 35–44 years, used amphetamine or methamphetamine, and were injecting drugs in the previous month had a significantly higher self-reported HIV prevalence and should be targeted for HIV diagnosis and linkage to anti-retroviral therapy (ART) services. Past studies of PWID in Kabul [3, 4] also reported a high prevalence of shared injection (33.6%), having bought sex from female sex workers (66%), having sex with another male (28%), and a high frequency of unsafe sex (66%) in 2009, a high frequency of injection (82.6%), and having been injected more than one year (56.3%) in 2012. Additionally, certain groups of male PWID, such as those who speak Pashto and those who actively inject drugs (reported their last injection during the month before the survey), had higher self-reported HIV prevalence. Screening and linkage to treatment for these groups can help them access and start HIV treatment. Language spoken may serve as a proxy for geographic origin or residence, potentially indicating regional differences in self-reported HIV prevalence, access to testing, awareness, and health service utilization. In our study, individuals from certain regions who spoke Uzbek might face higher vulnerability due to variations in HIV epidemic dynamics, stigma, or health system capacity for HIV testing and care.

We found that HIV testing among male PWID has increased significantly since the last study in Afghanistan in 2012. Using respondent driving sampling, a previous survey in 2012 in Afghanistan revealed that a much lower number of PWID had ever been tested for HIV [4]. Over several years, interventions such as the expansion of HIV testing sites [10], the addition of rapid HIV tests to needle and exchange programs [11], peer support [12], the leveraging of social networks to disseminate messages on HIV awareness [12, 13], the increase in awareness of HIV and the importance of HIV testing, especially among those at high risk, such as PWID [13], have led to improvements in the coverage of HIV testing programs in Afghanistan. However, there are still certain areas, such as Kanda, and certain subgroups, such as those who use opium, need to be targeted for HIV programs, as they have lower HIV testing rates. Partnerships with trusted community leaders through community-based interventions, such as home-based self-testing, mobile outreach, and hybrid approaches, can improve HIV testing in low-resource settings among priority populations, resulting in increased HIV testing [14].

Venue-based sampling, though commonly used for hard-to-reach populations like PWID, has inherent limitations that may introduce selection bias. It captures only individuals present at specific venues during sampling periods, excluding those who avoid these spaces due to stigma or other factors, leading to underrepresentation of certain subgroups. Additionally, sampling times may not reflect the diversity of the population, and individuals who frequent venues more often may differ in risk profiles or behaviors. These biases can affect the generalizability of our findings, particularly HIV prevalence estimates. Studies with responded-driven sampling [15] that enrolled PWID via networks or other novel sampling methods such as starfish sampling that combined venues and network enrolment strategies [16] can be useful to see if there was such a selection bias affected our findings.

Our study has three main limitations. First, we measured the HIV prevalence via self-reports among those who had ever been tested for HIV in the past; it is possible that some people did not report the truth about their HIV status (most likely not disclosing that they were HIV positive) or that their status has changed since the time they were tested for HIV. These factors might have resulted in an underestimation of the true HIV prevalence. Second, the participants in our study were recruited from several locations where PWID used to gather and see their peers; thus, we did not enroll those who did not visit such venues in our study. Finally, owing to changes in the health system and governance in Afghanistan and the COVID-19 disruption of services, our data on HIV testing and prevalence may not reflect the current situation in Afghanistan. A new survey of PWID is needed to obtain an updated situation and results.

Despite these limitations, our multicity study revealed some progress in HIV testing among one of the key populations, male PWID, at high risk for HIV in Afghanistan. The high self-reported HIV prevalence among this group also highlights the need for targeted screening and treatment programs for this group in several cities in Afghanistan. Targeted interventions such as expanding harm reduction services among this population can reduce the risk of HIV transmission among PWID in Afghanistan.

## Data Availability

The datasets used and/or analyzed in the current study are available from the corresponding author upon reasonable request.
